# Root Canal Treatment of a Hypertaurodont Mandibular Second Molar: A Case Report

**DOI:** 10.15171/joddd.2015.012

**Published:** 2015-03-04

**Authors:** Davood Jamshidi, Alireza Adl, Fereshte Sobhnamayan, Mehrdad Bolurian

**Affiliations:** ^1^Assistant Professor,Department of Endodontics, Faculty of Dentistry, GhazvinUniversity of Medical Science, Ghazvin, Iran; ^2^Assistant Professor, Department of Endodontics, Faculty of Dentistry, Shiraz University of Medical Science, Shiraz, Iran; ^3^Assistant Professor, Department of Endodontics, Faculty of Dentistry, Ardebil University of Medical Science, Ardebil, Iran

**Keywords:** Pulp chamber, root canal, taurodontism

## Abstract

Taurodontism is a morphologic feature of generally multi-rooted teeth with large pulp chambers and shortened roots. A case of endodontic treatment in a 24-year-old male with the chief complaint of swelling and pain in the right mandibular region is described. Periapical lesion was present in the radiographic examination of hypertaurodont second mandibular molar.Four root canals were detected with an endodontic microscope. The canals were prepared and obturated with lateral condensation technique in the second appointment. The patient was asymptomatic in the 18-month follow-up.

## Introduction

Comprehensive knowledge of tooth anatomy and root canal morphology is crucial in success of root canal treatment. The term “taurodontism” was first introduced in 1908 and is defined as a morphologic change of generally multi-rooted teeth with large pulp chamber which its pulpal floor and furcation is apically displaced.^1^This anomaly is the result of Hertwig's epithelial sheath inadequacy to invaginate at the proper horizontal level.^2^ Taurodontisms can be seen unilaterally or bilaterally, in any quadrants, and in both permanent and deciduous dentition.^3,4^ Review of literature shows a wide range in the prevalence of taurodontisms in different populations; however, one study showed the prevalence of this anomaly to be 5.5%in the south of Iran.^5^According to the furcation level, taurodontism has been classified into mild “hypotaurodontism,”moderate “mesotaurodotism,” and severe“hypertaurodontism.”^6^ Taurodontism has appeared as an isolated anomaly most of the times, but association with several syndromes, familial inheritance, and genetic malformation has been reported.^2,7^

## Case Report 

**Figure 1.**
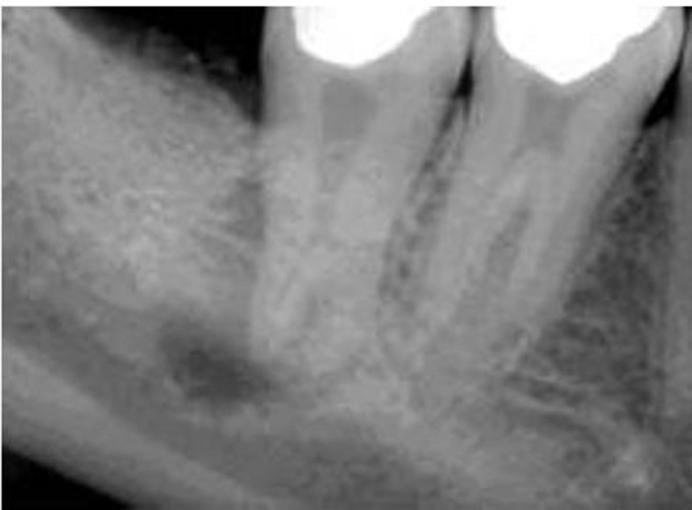
Preoperative radiograph of right mandibular second molar with hypertaurodontism and periapcial radiolucency.

**Figure 2.**
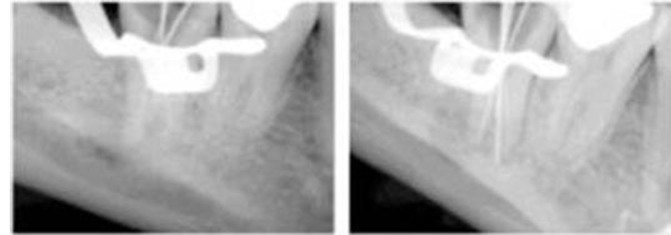
Radiograph taken for confirming the working length of endodontic treatment.

A 24-year-old male patient with a non-contributory medical history was referred to Shiraz University of Medical Sciences Department of Endodontics. The chief complaint of the patient was swelling in the right mandibular region. Clinical examination revealeda deep amalgam restoration in the right mandibular second molar and a firm swelling in submandibular region. Performing vitality tests, complete necrosis was confirmed. Preoperative radiograph showed a priapical radiolucency of the mentioned tooth. The tooth revealed the characteristics of hypertarodontia in the radiograph(Figure 1). After achieving anesthesia with 2% lidocaine and 1:80000 epinephrine (Daroupakhsh, Tehran, Iran), the tooth was isolated with a rubber dam and access cavity was prepared. Detection of the root canals deemed difficult and was performed with an endodontic microscope (OPMI Pico Dental Microscope; Zeiss, Oberkochen, Germany).Four root canals were detected. Working length was determined with an electronic apex locator (Raypex 5, VWD GmbH, Munich, Germany) and confirmed with k-files (Dentsply-Maillefer, Ballaigues, Switzerland) and radiography (Figure 2).

**Figure 3.**
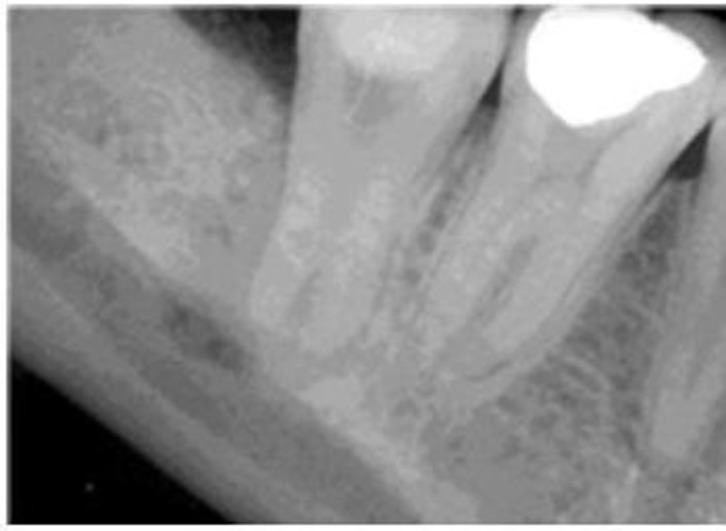
Radiographic image taken after placement of intracanal medication at the end of first visit.

Individual canal instrumentation was performed with ProTaper (Dentsply-Maillefer, Ballagius, Switzerland) rotary Ni-Ti files, S1to F2. Canals were irrigated with 2.5%sodium hypochlorite. Calcium hydroxide paste with normal saline was placed as an intracanal medicament with a lentulospiral and the access cavity was sealed with a temporary filling material (Cavisol; Golchai CO., Tehran, Iran; Figure 3). Incision and drainage was performed in the same session.

**Figure 4.**
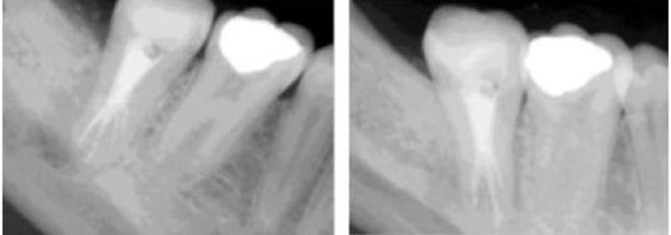
Immediate post-op radiographic images show obturation of four canals.

**Figure 5.**
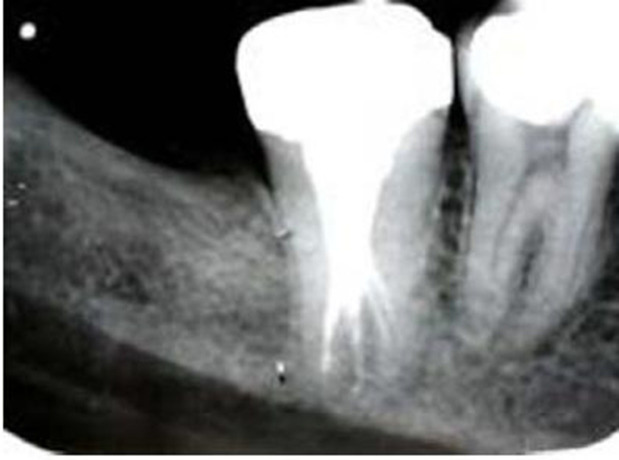
Radiographic evaluation of treated tooth at 18-month follow-up. The periapical radiolucency has disappeared. The patient is asymptomatic.

Ten days later at the second appointment, the patient was asymptomatic. Root canals were irrigated with sodium hypochlorite to remove calcium hydroxide and obturation was performed using cold lateral condensation of gutta-percha (Dentsply-Maillefer, Ballagius, Switzerland) and AH-26sealer (Dentsply DeTrey, Konstanz, Germany). Access cavity was sealed with the same temporary filling material and the patient was referred for final restoration (Figure 4). The patient was asymptomatic in the18-month follow-up, and no radiolucency was seen in the radiograph (Figure 5).

## Discussion

Prevalence of taurodontism has been reported to be 5.5% in the south of Iranwith67% of taurodonts being hypotaurodont, 31% mesotaurodont, and 2% hypertaurodonts.^5^ Females had higher prevalence of taurodontism than men and mandibular second molar was the most affected tooth.^5^Clinically, a taurodont cannot be differentiated from a normal tooth because its body and roots lie below the alveolar margin.^8^The radiographic feature of taurodontism is extension of the pulp chamber into the elongated body of the tooth, shortened roots and root canals, and location of furcation near the root apices.^6^

According to the mentioned classification,^6^ present case can be classified as hypertaurodont with four short root canals.As for difficulty of finding and preparingthe canals of this tooth,the use of magnification was crucial. Taurodontism complicates the endodontic procedure due to an abnormal root canal system that may disturb the location of the root orifices, thereby increasing the difficulty of instrumentation and obturation.^9^ The complexity of root canal system in taurodonts makes sufficient instrumentation nearly impossible; therefore, sodium hypochlorite can improve root canal cleaning because of its ability to dissolve the remaining pulp tissues.^10^

## Conclusion

Proper clinical and radiographic examination is a key factor forsuccessful endodontic treatment. Endodontic treatment of taurodonts is often challenging and needs more time than usual treatment. Careful evaluation for detecting additional canals is crucial due to potential of an abnormal root canal system.

## References

[1] Saini T, Wilson CA (1990). Taurodont molars: review of literature and radiological features. Saudi Dent J.

[2] Mena CA (1971). Taurodontism. Oral Surg Oral Med Oral Pathol.

[3] Sert S, Bayirli G (2004). Taurodontism in six molars: a case report. J Endod.

[4] Chaparro González NT, Leidenz Bermudez JS, González Molina EM, Padilla Olmedillo JR (2010). Multiple bilateral taurodontism. A case report. J Endod.

[5] Bronoosh P, Haghnegahdar A, Dehbozorgi M (2012). Prevalence of taurodontism in premolars and molars in the south of Iran. J Dent Res Dent Clin Dent Prospects.

[6] Neville BW, Damm DD, Allen CM, Bouquot JE,  eds  eds (2009). Oral and Maxillofacial Pathology, 3rd ed.

[7] Barker BC (1971). Taurodontism: the incidence and possible significance of the trait. Aust Dent J.

[8] Mangion JJ (1962). Two cases of taurodontism in modern human jaws. Br Dent J.

[9] Marques-da-silva B, Baratto-Filho F, Abuabara A, Moura P, Losso EM, Moro A (2010). Multiple taurodontism: the challenge of endodontic treatment. J Oral Sci.

[10] Prakash R, Vishnu C, Suma B,  Velmurugan  Velmurugan, Kandaswamy D (2005). Endodontic management of taurodontic teeth. Indian J Dent Res.

